# Design of Multiplex Lateral Flow Tests: A Case Study for Simultaneous Detection of Three Antibiotics

**DOI:** 10.3390/bios10030017

**Published:** 2020-02-27

**Authors:** Anastasiya V. Bartosh, Dmitriy V. Sotnikov, Olga D. Hendrickson, Anatoly V. Zherdev, Boris B. Dzantiev

**Affiliations:** A.N. Bach Institute of Biochemistry, Research Center of Biotechnology of the Russian Academy of Sciences, Leninsky prospect 33, Moscow 119071, Russia; bartoshlab@yandex.ru (A.V.B.); sotnikov.dv@gmail.com (D.V.S.); odhendrick@gmail.com (O.D.H.); zherdev@inbi.ras.ru (A.V.Z.)

**Keywords:** multiparametric assay, rapid tests, immunochromatography, antibiotics, non-equilibrium interactions

## Abstract

The presented study is focused on the impact of binding zone location on immunochromatographic test strips on the analytical parameters of multiplex lateral flow assays. Due to non-equilibrium conditions for such assays the duration of immune reactions influences significantly the analytical parameters, and the integration of several analytes into one multiplex strip may cause an essential decrease of sensitivity. To choose the best location for binding zones, we have tested reactants for immunochromatographic assays of lincomycin, chloramphenicol, and tetracycline. The influence of the distance to the binding zones on the intensity of coloration and limit of detection (LOD) was rather different. Basing on the data obtained, the best order of binding zones was chosen. In comparison with non-optimal location the LODs were 5–10 fold improved. The final assay provides LODs 0.4, 0.4 and 1.0 ng/mL for lincomycin, chloramphenicol, and tetracycline, respectively. The proposed approach can be applied for multiplexed assays of other analytes.

## 1. Introduction

The development of analytical techniques for the simple and rapid detection of various compounds is currently a task in high demand. Immunochromatographic assays (ICAs) are among the most efficient approaches for this purpose. They are actively applied for detection of pathogens, biomarkers of infection diseases and functional disorders, hormones and other bioregulators, toxic contaminants of food stuffs and environmental samples [1,2,3,4,5]. The advantage of ICA lies in the fact that all required reactants are already applied on the test strip before its use. Upon contact of the strip with the tested liquid sample, the liquid removes the applied labeled antibodies, which, during the process of moving along the membrane, specifically bind to the detected analyte in the sample and are fixed on the test zone of the strip. ICA is thus a simple technique with minimal sample preparation requirements, and the results are obtained in 5–15 min. The low cost of ICA in comparison with other analytical methods determines its potential as a competitive tool for primary mass screening.

Due to the increased number of controlled compounds in medical diagnostics, food safety and ecological monitoring, there is a need for multiplex test systems [6,7]. The location of several binding lines with reactants of different specificity is the best technological solution for multiplex ICA [8,9,10,11]. Such tests for 2–8 analytes are widely used to determine antibiotics in milk, mycotoxins in grain, etc. and described in a series of recent publications [12,13,14,15,16].

However, the integration of several successfully developed monoplex ICAs into one multiplex ICA is associated with several essential problems. The key question in this work is how to ensure the sensitivity of all monoplex ICAs. By combining several reactants in one test strip, we are forced to change the position of the binding zones for different analytes and, accordingly, the conditions of their interactions. First of all, there is issue of the duration of the interaction in solution, when labeled antibodies and compounds of the sample are moved to the corresponding binding zone. This factor is important, as is the fact these interactions often do not reach equilibrium, and the degree of equilibrium reached is critical for the number of complexes formed and detected. Also the speed of flow movement along the membrane slows down in accordance with the increased distance from the start. This speed essentially affects the sensitivity of the test and the location of the test zone in the vicinity of the control zone and at the end of the working membrane is commonly considered as the preferable choice [17]. However, this demand cannot be simultaneously reached for all compounds in a multiplex test. Besides, the concentrations of interacting reactants change as a result of dilution of their initial volumes when the flow moves along the test strip [17,18]. This influences not only the chemical equilibrium in solution, but also the efficiency of label binding when they reach the test zone, so not only the detection limit, but also the intensity of coloration (and, thus the reliability of visual estimation of the assay results) may vary significantly in multiplex tests.

All these factors are stated as important ones in theoretical studies and common guides. However, the published works about multiplex ICA usually do not consider them when choosing a test system design. As a result of this, the transition from monoplex competitive ICAs to multiplex competitive ICA is often associated with losses in coloration of binding zones and a shift in the detection limit. Despite the availability of a number of published developments without indicating changes in the location of the tests zones, the risks of such problems at a laboratory level may be considered as significant, so our work is focused on explicit consideration of changes in the assay parameters during the transition to multiplex ICA and finding solutions to estimate key parameters of immune reactants and finding the best order of their deposition on the test strip. The quantity of the possible orders increases dramatically with the growth of the quantity of test zones (N) and is equal to N! (i.e., six for three analytes, 24 for four analytes, 120 for five analytes, etc.), so extensive characterization of all possible variants becomes an extremely time-consuming task and should be replaced by simple techniques for the assessment of individual reactants. The consideration of triplex ICA seems to be an informative study for such a test of reagents and the identification of their significant characteristics, because explanation of the differences between six design variants in the case of extensive characterization is already becomes very onerous.

Our development for the assay of three antibiotics is important for food safety and medicine, where multiplexed tests are especially relevant. Due to the widespread use of antibiotic therapy, bacterial resistance has become a reason for more careful monitoring of their levels in the course of therapy. Tetracycline, chloramphenicol and lincomycin are widely used antibiotics in the treatment of diseases caused by microorganisms. However, their improper use has provoked massive resistance to them in many microorganisms. Resistance to tetracycline is described for strains of *Pseudomonas aeruginosa, Proteus*, *Staphylococcus aureus* (MRSA) and *Streptococcus pneumoniae* [19], to chloramphenicol for *Bartonella* and *Staphylococcus* [20], and to lincomycin for *Kocuria kristinae, Sphingomonas paucimobilis, Pantoea*, *Staphylococcus vitulinus*, *Clostridium*, etc. [21,22]. Individual correction of antibiotics dosage decreases the level of antibiotic-resistant bacteria in the intestines [23]. These reasons determine the practical importance of multitests for antibiotics not only for food safety control (where successfully commercialized tests are available) but also for planning efficient individual therapy.

The study and the proposed recommendations are addressed to competitive LFIA, but the problem of integration several monotests into one multiplex test on one test strip requires solutions for sandwich LFIA, serodiagnostic LFIA, and other formats of LFIA too. However, due to the different composition and features of sequential formation for colored complexes detected in the test zone other formats need additional studies.

## 2. Materials and Methods

### 2.1. Reactants

Tetracycline (TET) base was from Applichem GmbH (Darmstadt, Germany), chloramphenicol (CAP) base and lincomycin (LIN) base were from Sigma-Aldrich (St. Louis, MO, USA). Mouse anti-tetracycline, anti-chloramphenicol and anti-lincomycin monoclonal antibodies, as well as conjugates of TET, CAP and LIN with bovine serum albumin (BSA) were purchased in Eximio Biotec (Wuxi, China). The used antibodies did not interact with antibiotics from other chemical classes, as we have demonstrated earlier for individual ICAs of tetracycline [24], chloramphenicol [25] and lincomycin [26].

Goat anti-mouse (anti-species) polyclonal immunoglobulins G (IgG) and mouse IgG were purchased in Arista Biologicals (Allentown, PA, USA). Compounds for the preparation and storage of gold nanoparticles—chloroauric acid, sodium citrate, sodium azide and Tween 20—were from Sigma-Aldrich. BSA was from Boval Biosolutions (Cleburne, TX, USA). Saccharose, Pharma Sugar Raffine, was from Cristalco (Paris, France). Tris, NaCl, K_2_PO_4_ and KOH were from Chimmed (Moscow, Russia).

All the solutions were prepared using deionized water (18 MΩ × cm at 25 °C) obtained with the use of Milli-Q Simplicity system (Millipore, Billerica, MA, USA). Working nitrocellulose membrane CNPC 15 was from MDI Membrane Technologies (Ambala Cantt, India). Glass-fiber membrane was from Millipore, absorbance membrane CF5 was from Whatman (GE Healthcare Bio-Sciences, Marlborough, MA, USA).

### 2.2. Synthesis of Gold Nanoparticles and Their Conjugation with Antibodies

Gold nanoparticles were synthesized according to standard protocol for 30 nm nanoparticles as in our previous work [27]. First, 0.1 mL of 10% HAuCl_4_ was added to 97.5 mL of water filtered via Milli-Q membrane filter, 0.18 μm pore size, followed by boiling. After that 1.5 mL of 1% sodium citrate was added and the resulting mixture was boiled for 25 min, cooled and then stored at 4 °C. Further details of this method can be found in [28].

Goat anti-mouse IgG dialyzed against 10 mM Tris-HCl buffer (pH 8.6) was diluted by the pre-adjusted to pH of 8.5 gold nanoparticles by 0.2 M K_2_CO_3_ solution. For this, 8 mL of gold nanoparticles was added to 150 μL IgG solution (1000 μg/mL) to the glass flask. The solution was incubated with stirring for 45 min at room temperature. 200 μL of 10% aqueous solution of BSA was added to the mixture with followed incubation under stirring for 15 min at room temperature. The obtained conjugate was separated from unbound IgG by centrifugation at 10,000 *g* at + 4 °C for 15 min with decanting of the supernatant liquid. Then 1 mL of 50 mM potassium phosphate buffer, pH 7.4 with 0.1 M NaCl (PBS) containing 0.25% BSA, 0.25% Tween 20, 1% saccharose and 0.05% NaN_3_ was added to the precipitate. The composition of the buffer was based on the previous studies described in [24]. An optical density of the obtained conjugate was established using a Libra S60 spectrophotometer (BioChrom, Cambridge, UK).

### 2.3. Preparation of the Test Strips

The LIN-BSA, TET-BSA and CAP-BSA conjugates (for the test zone) and mouse IgG (for the control zone) were applied to the working nitrocellulose membrane in an amount of 0.1 μL per 1 mm. The concentrations of LIN-BSA, TET-BSA and CAP-BSA conjugates were chosen on the base of our previous studies to provide sufficient coloration in the absence of analyte [24,25,26] and were equal to 1, 0.5 and 0.5 mg/mL respectively. The difference in the chosen concentrations for these commercial conjugates may be caused by their composition and/or sorption properties. The mouse IgG concentration was 0.05 mg/mL.

The anti-species IgG conjugate in the PBS containing 0.25% BSA, 0.25% Tween 20, 1% saccharose was applied to the glass-fiber sample membrane (width 5 mm) in 32 μL/cm.

After applying the reagents, the membrane was dried for 10 h at 37 °C. Next, we took the working membrane on the backing pad (connected by the manufacturer), peeled off the protective sticker from the bottom of the substrate, and applied (with a slight pressure) the glass-fiber membrane to the adhesive part. Further, the protective sticker was peeled off from the upper adhesive part of the backing pad and an absorbent membrane was applied (with a slight pressure).

The resulting master sheet, using an automatic guillotine cutter, cut the received test strips 3.3 mm wide. Test strips together with a desiccant (0.6 g of silica gel in bags) were sealed in a plastic foil pact and sealed. Cutting and packaging was carried out at 20–22 °C in a special room with a relative humidity of not more than 30%.

### 2.4. Performing Lateral Flow Immunoassay

Test strips were brought to room temperature and immersed into the samples with various concentrations of antibiotics in 50 mM phosphate buffer, pH 7.4, with 0.25% Tween 20 [24] for 7 min.

### 2.5. Data Processing

After the assay, the strips were scanned in a flatbed scanner (Canon Lide 90, Canon, Tokyo, Japan) with a resolution of 600 dpi without applying modes for contrast or color correction. The intensities of coloration were quantified using the Total Lab software package (TotalLab, Newcastle upon Tyne, UK).

The dependence of the color intensity from antibiotics concentration in the sample was determined with the Origin 7.5 software (Origin Lab, Northampton, MA, USA). The dependence was approximated using the four-parameter sigmoid function, and the instrumental limit of detection (LOD) and IC_50_ were calculated.

To obtain a cut-off level of coloration (visual LOD), we estimated the instrumentally registered color intensity in the test zone, which corresponds to the appearance of visible color. The signal processed by the Total Lab corresponded to this appearance was equal to 25 arbitrary units. Using sigmoidal fittings of the dependences of the color intensity from concentrations of TET, CAP and LIN, we calculated concentrations of these antibiotics that accord to the indicated above cut-off levels of coloration.

## 3. Results and Discussion

The advantages of indirect labeling in competitive ICA has been shown in our previous studies [29,30,31,32] and summarized in [33]. They consist in prevention of non-productive binding of analyte with specific antibodies without changes of detected signals and possibility to vary independently concentrations of nanosized label (increasing for higher signal) and specific antibodies (decreasing for more efficient competition). In our study the indirect labeling gives additional possibility to use the same conjugate to generate signals in all analytical zones and thus exclude potential difference in immobilization of different specific antibodies on gold nanoparticles (that could occur for competitive LFIA with direct conjugation of specific antibodies).

**Figure 1:**
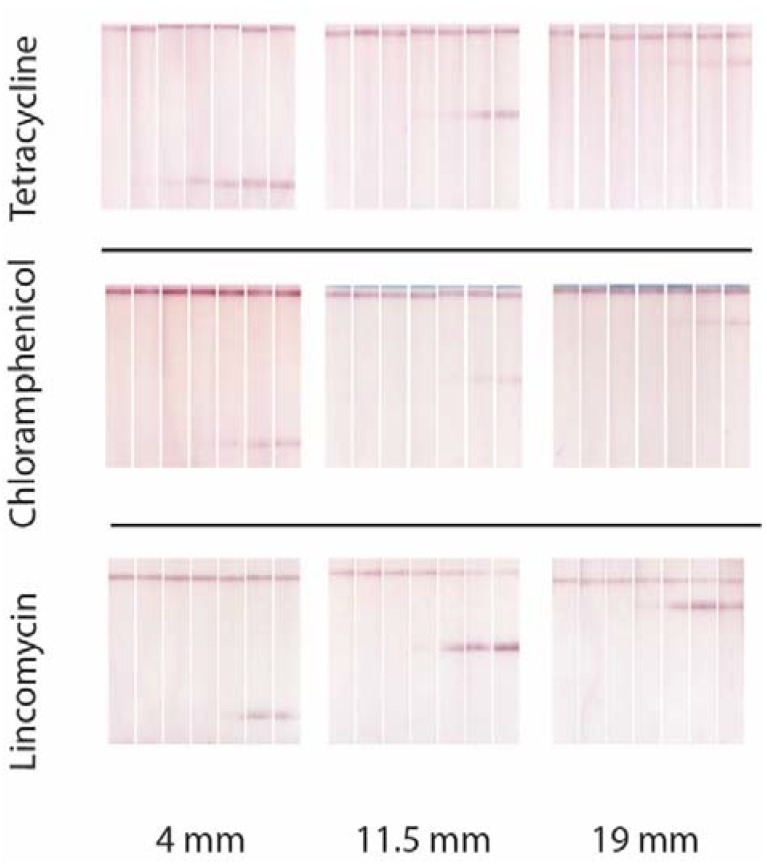
Test strip images after analysis of antibiotics-contained serum samples for different location of test zones. Test strips are arranged from left to right in accordance with the antibiotics concentration (100, 33, 11, 3.7, 1.2, 0.4 and 0 ng/mL).

**Figure 2:**
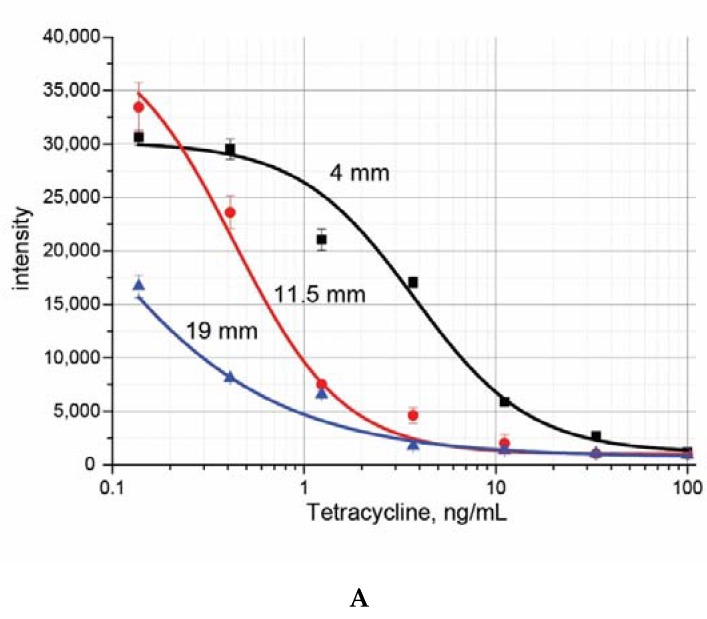

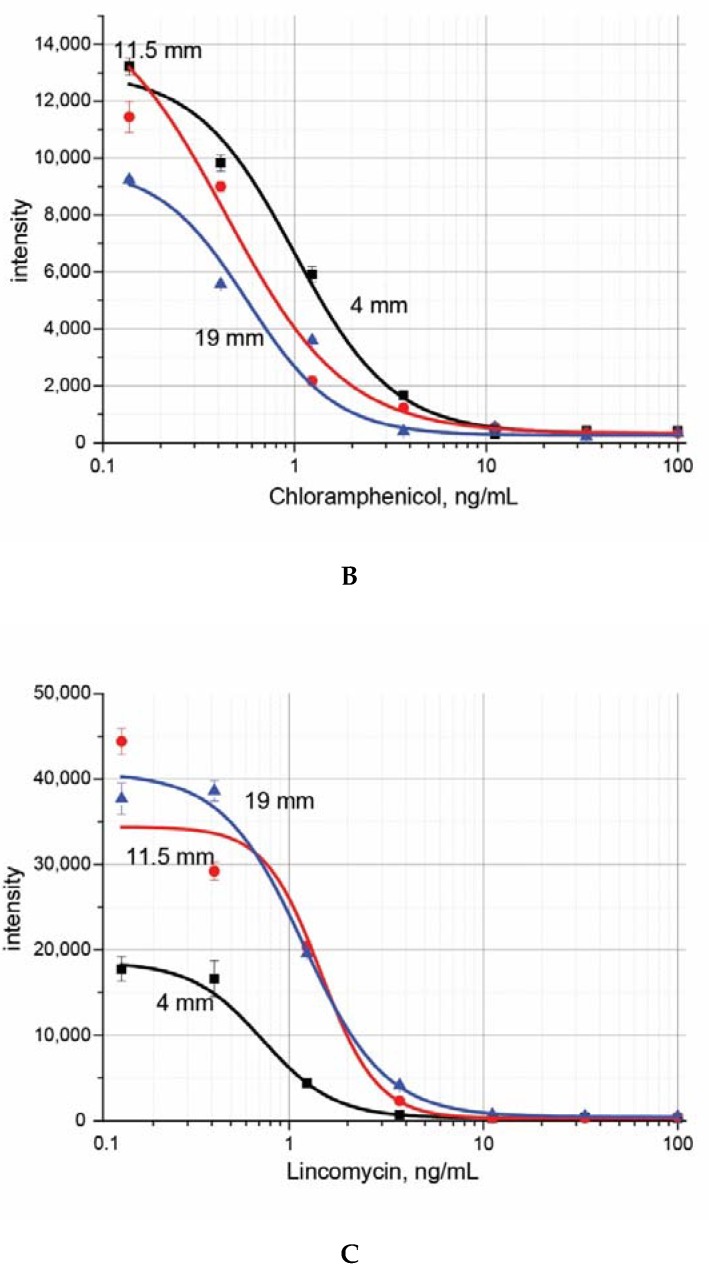
Calibration curves for monoplex lateral flow immunoassays of tetracycline (**A**), chloramphenicol (**B**), and lincomycin (**C**) for different location of test zones, n = 3.

Location of the test line on the membrane influence analytical parameters even for monoplex immunochromatography [17]. To characterize the corresponding features for all immunoreactants used in our triplex test, we placed the test zone for each analyte in three positions: 4, 11.5 and 19 mm. The obtained results for three antibiotics, tetracycline, chloramphenicol, and lincomycin, are given in Figure 1, and comparisons of calibration curves are integrated in Figure 2.

The obtained results show how changes of the intensity of coloration and shift of the working range for each set of immunoreactants. In the case of tetracycline the increased distance of flow leads to better sensitivity, but for the 19 m run this improvement is associated with significantly decreased coloration. In the case of chloramphenicol changes in location and amplitude of the calibration curves are minimal. The system for lincomycin demonstrates much better sensitivity and appropriate intensity of signal for minimal distance of the test zone from the beginning. Thus, the LOD may be change up to 10 times by a simple change of the zone location.

**Figure 3:**
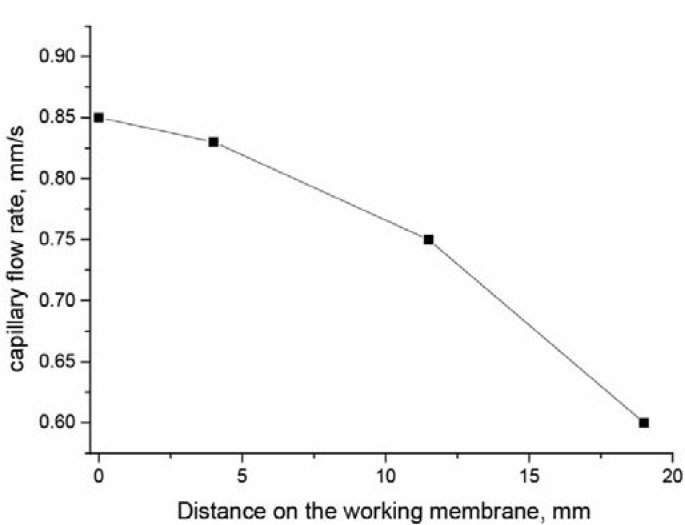
Change in the rate of capillary flow in the course of liquid movement along the working membrane of the test strip.

Considering mechanisms causing the demonstrated changes, we should two key factors that affect the flow rate of reagents: (i) size and surface properties of the pores of the working membrane and (ii) the location of the test zone. To estimate these changes, we recorded the flow rate in the course of reactants movement using an iPhone camera. We compared the coloration profile in 1 sec intervals, indicating the first image with reliable increase of the coloration at the test zone and calculatinf the flow rate as a ratio of the distance covered by the colored reagent to this time. The obtained data are summarized in Figure 3. The reagent flow reached the first test zone (4 mm) at an average rate of 0.83 mm/s. For the second test zone (11.5 mm) the average rate was 0.79 mm/s, and for the third zone (19 mm) it was equal to 0.60 mm/s. For different samples, the flow rate time is approximately the same. The edge of the working membrane in working buffer, milk and diluted serum (1:1) flow reaches in 27, 28 and 29.5 s, respectively.

The exponential decrease of the flow rate in the accordance with the increased distance from the start of the flow was earlier demonstrated for immunochromatographic processes [17,18]. This phenomenon causes the influence of the test zones location on the assay sensitivity. If the line with the applied reagent is located at a higher distance from the start, the front of the liquid with the analyte passes more slowly through this line and the quantity of bound complexes formed is increased (more intense coloration). An additional key factor is the time necessary to reach equilibrium for the interactions of reactants in flow. In the case of more affine antibodies the final quantity of immune complexes is formed at the starting part of the working membrane, and additional elongation of the way does not cause improvements in sensitivity. Contrary, for low-affine interactions the choice of maximal distance for the test zone is reasonable. Thus, the preliminary controlled influence of the test zone location on the LOD and intensity of coloration allows separating immunoreactants for multiassay to several groups, with preferable minimal distance of the test zone from the starting point, the preferable maximal distance and without influence of the distance on analytical parameters.

**Figure 4:**
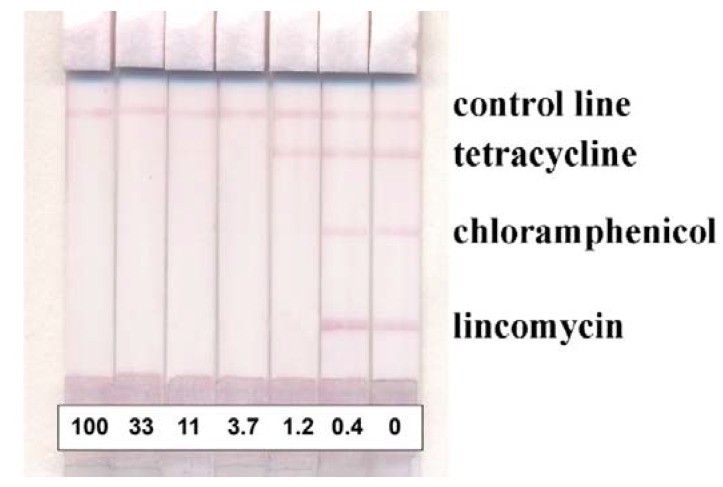
Appearance of the developed multiplex tests for three antibiotics. Test strips are arranged in accordance with the antibiotics concentrations in ng/mL.

Basing on these rules and the found difference in immunoreactants properties, we have designed a multiplex system where the position of the test zones guarantees a high-quality assay (low LOS and acceptable intensity of coloration). We placed lincomycin at the 4 mm position, chloramphenicol at the 11.5 mm position and tetracycline at the 19 mm position. The test system demonstrates the instrumental detection limits of 0.4 ng/mL, 0.4 ng/mL and 1.0 ng/mL for lincomycin, chloramphenicol and tetracycline, respectively (see Figure 4 and Table 1).

We believe that for some immunoreactants, the location of the test zone at the beginning could be critical due to slow immune interactions in solution and the limited time of the flow movement along the test zone. The location of the test zone as far as possible from the start point may eliminate these problems. Alternatively, some other reactants probably provide efficient immune binding both in solution and with immobilized reactants independently on the location of the test zone (see lincomycin example in our study).

The consideration of k_on_ and k_off_ rates and the estimation of time for reaching chemical equilibrium will be the strong rationale for choice of the test zones locations. However, the proposed approach with screening comparison of several locations for individual reactants provides simple testing without involvement of additional time- and labor consuming techniques.

It should be noted additionally that tested matrixes may influence significantly on the capillary flow rate and reaching chemical equilibrium in the course of lateral flow processes, so the testing of immune reactants should be implemented in liquid media providing the same viscosity and flow rate as the real samples.

## 4. Conclusions

In the development of multiplex lateral flow tests, we have used data on changes in sensitivity depending on the location of the test zones. We showed that a simple shift of these zones to the region of lower speeds results in an order of magnitude more sensitive assay. On the other hand, for some immunoreactants chemical equilibrium of their interactions is reached rapidly, and so they could be located next to the starting region of the working membrane. Using the knowledge about the change in the capillary flow velocity, we constructed a multiplex test for three antibiotics where the test zones are located at a distance that provides the lowest detection limits. Knowledge of these patterns can be applied in the development of various multiplex tests including ones with four or more test zones and will provide high-sensitive assays for all analytes without laborious screening of all possible immunoreactant location variants for the working membrane.

## Figures and Tables

**Table 1:** Analytical parameters (n = 3) of the developed multiplex test for three antibiotics.

	LOD Instrumental, ng/mL	IC_50_, ng/mL	LOD Visual, ng/mL
Lincomycin	0.5 ± 0.4	0.8 ± 0.5	1.3 ± 0.3
Chloramphenicol	0.4 ± 0.2	0.7 ± 0.3	1.2 ± 0.2
Tetracycline	1.1 ± 0.4	1.7 ± 0.4	2.7 ± 0.5
